# Age-related differences in the intrinsic connectivity of the hippocampus and ventral temporal lobe in autistic individuals

**DOI:** 10.3389/fnhum.2024.1394706

**Published:** 2024-06-13

**Authors:** Lang Chen, Meghan Abate, Mackenzie Fredericks, Yuanchun Guo, Zhizhen Tao, Xiuming Zhang

**Affiliations:** ^1^Department of Psychology, Santa Clara University, Santa Clara, CA, United States; ^2^Neuroscience Program, Santa Clara University, Santa Clara, CA, United States; ^3^Department of Counseling Psychology, Santa Clara University, Santa Clara, CA, United States

**Keywords:** hippocampus, ventral temporal lobe, autism spectrum disorder, memory, functional connectivity

## Abstract

**Introduction:**

Although memory challenges in autistic individuals have been characterized recently, the functional connectivity of the hippocampus and ventral temporal lobe, two structures important for episodic and semantic memory functions, are poorly understood in autistic individuals. Moreover, age-related differences in the functional connectivity associated with these two memory networks are unrevealed.

**Methods:**

The current study investigated age-related differences in intrinsic connectivity of the hippocampal and ventral temporal lobe (vTL) memory networks in well-matched ASD (*n* = 73; age range: 10.23–55.40 years old) and Non-ASD groups (*n* = 74; age range: 10.46–56.20 years old) from the open dataset ABIDE-I. Both theory-driven ROI-to-ROI approach and exploratory seed-based whole-brain approach were used.

**Results and discussion:**

Our findings revealed reduced connectivity in ASD compared to Non-ASD peers, as well as an age-related reduction in the connectivity of hippocampal and vTL networks with triple networks, namely, the default mode network (DMN), the central executive network (CEN), and the salience network (SN), potentially underpinning their challenges in memory, language, and social functions. However, we did not observe reliable differences in age-related effects between the ASD and Non-ASD groups. Our study underscores the importance of understanding memory network dysfunctions in ASD across the lifespan to inform educational and clinical practices.

## 1 Introduction

Individuals diagnosed with autism spectrum disorder (ASD) often experience cognitive and social difficulties that significantly impact their daily functioning (Di Martino et al., 2009; Zablotsky et al., 2015; Danker et al., 2016; Cai et al., 2020). In addition to commonly recognized symptoms such as repetitive behaviors, restricted interests, and challenges in socio-communicative functions, autistic individuals frequently exhibit atypical memory functions (Lynn et al., 2018; Desaunay et al., 2020; Liu et al., 2023; Chen et al., 2024). Although their behavioral profiles of memory difficulties are now well characterized in the literature (Desaunay et al., 2020), there remains a notable gap in our understanding of the neural correlates underlying their memory challenges (Bowler et al., 2011; Cooper et al., 2017; Banker et al., 2021; Desaunay et al., 2023). Furthermore, little attention has been given to investigating how these neurobiological underpinnings change over the lifespan, particularly regarding memory functions in autistic individuals. Thus, this study aims to elucidate the age-related alterations in intrinsic brain connectivity associated with memory functions in autistic individuals, compared to their non-autistic counterparts.

Among the various types of memory functions, challenges in episodic memory have received considerable attention in the research on autistic individuals (Lind and Bowler, 2010; Griffin et al., 2022). Studies have consistently indicated that autistic individuals often exhibit challenges in episodic memory compared to their Non-ASD peers (Boucher et al., 2012; Boucher and Anns, 2018). For instance, one study revealed that a group of autistic adults, with an average age of 36, showed diminished relational encoding abilities, consequently contributing to their decreased recollection (Gaigg et al., 2015). Similarly, investigations have suggested that episodic memory difficulties may manifest in autistic children as early as the age of 6 (Naito et al., 2020). Given the established role of the hippocampus in episodic memory (Scoville and Milner, 1957; Bird and Burgess, 2008; Voss et al., 2017), previous literature also explored the role of the hippocampus in ASD. Studies have observed increased activation patterns in the hippocampus during episodic memory tasks in autistic individuals (Hogeveen et al., 2020) and reported reduced functional connectivity of the hippocampus compared to Non-ASD (Cooper et al., 2017; Hogeveen et al., 2020). Some studies even revealed increased hippocampal activations in ASD when they were exposed to sensory stimuli (Green et al., 2013). Collectively, these findings suggest that hippocampal dysfunction likely contributes to the challenges in episodic memory of autistic individuals.

Considering its crucial role in episodic memory functions, numerous studies have investigated the structure and function of the hippocampus in ASD across different developmental stages (Richards et al., 2020; Xu et al., 2020). In early childhood (24–72 months), research suggests that male individuals with ASD have a larger right hippocampus compared to non-autistic peers (Reinhardt et al., 2020). Similar findings have been reported for autistic individuals in late childhood (7.5–18.5 years old; Schumann et al., 2004) and young adulthood (6.5–27 years old; Xu et al., 2020). However, studies focusing on older adult groups (mean age ≥ 50 years old) have indicated smaller hippocampal volumes in ASD and a faster rate of reduction compared to the matched Non-ASD (Braden et al., 2017; Van Rooij et al., 2018; Pagni et al., 2022). Despite extensive research on resting-state functional connectivity in ASD (Hull et al., 2017), there is limited understanding regarding the resting-state functional connectivity of the hippocampus in ASD and relevant age-related effects. Some studies have reported greater connectivity of subcortical regions, including the hippocampus, only in young autistic children (Wiggins et al., 2011; Nomi and Uddin, 2015; Liu et al., 2023). Meanwhile, one study noted reduced functional connectivity of the hippocampus in young autistic adults compared to non-autistic individuals (Pereira et al., 2018). Therefore, a more comprehensive analysis is needed to examine the age-related differences in resting-state hippocampal connectivity across a broader age range beyond children and young adults.

In addition to episodic memory, aging in typically developing (TD) populations is often linked with semantic memory decline (Nilsson, 2003). While the neurobiological underpinnings of episodic memory are predominantly rooted in the medial temporal lobe (Voss et al., 2017), semantic memory relies on a distributed brain network with a critical hub in the ventral part of the temporal lobe (vTL), especially its anterior segment (Rogers et al., 2004; Binney et al., 2010; Visser et al., 2012; Lambon Ralph et al., 2016; Chen et al., 2017). For instance, studies have consistently demonstrated that profound semantic memory deficits in semantic dementia coincide with atrophy in the bilateral ventral temporal lobes (Hodges et al., 1992; Patterson et al., 2006; Chen and Rogers, 2014; Lambon Ralph et al., 2016). Additionally, research has highlighted a significant association between reduced lateral temporal surface area and aging, further suggesting the important role of the vTL in memory over aging (Hogstrom et al., 2013).

In contrast to episodic memory, previous studies indicated that semantic memory was preserved in autistic individuals (Coderre et al., 2017; Gladfelter and Goffman, 2018), leading to a lack of research on semantic memory or the vTL in ASD. However, recent behavioral studies have shown that ASD participants generally exhibit lower semantic fluency compared to TD, suggesting challenges in utilizing semantic knowledge for language functions to some extent (Ehlen et al., 2020; Foldager et al., 2023). Neuroimaging studies on vTL changes over aging in ASD are quite limited. Only a couple of studies suggested that the temporal cortex tends to be thinner in autistic individuals over aging (Wallace et al., 2010), and a greater local functional connectivity in the right temporal pole was observed in adults but not in children with ASD (Dajani and Uddin, 2016). Consequently, there is a pressing need for a comprehensive characterization of vTL-based connectivity for semantic memory across different age groups in ASD, particularly in comparison to the hippocampal network underlying episodic memory.

In sum, the present study aimed to address significant gaps in the literature by investigating age-related differences in intrinsic connectivity within the hippocampal memory network (related to episodic memory) and the ventral temporal lobe memory network (related to semantic memory) in ASD compared to Non-ASD populations. First, we employed a theory-driven network approach to examine the intrinsic connectivity of hippocampal and vTL nodes with brain regions that were shown to be significant in ASD. Specifically, our theory-driven network analysis would focus on brain regions in the Default Mode Network (DMN), Central Executive Network (CEN), and Salience Network (SN), known as the triple networks associated with neural dysfunctions in ASD (Uddin et al., 2011; Menon, 2018). Given the findings in the literature, we hypothesized that ASD would show aberrant functional connectivity between hippocampal and vTL memory nodes with brain regions in triple networks compared to Non-ASD. More importantly, these connectivity patterns would be associated with their age, and an Age-by-Group interaction could be observed in some connectivity. Next, we utilized a seed-to-whole-brain approach to explore the atypical functional connectivity of hippocampal and vTL nodes with brain regions outside the triple networks in ASD compared to Non-ASD. Then, we explored whether these atypical patterns of connectivity between ASD and Non-ASD would show age-related differences. By addressing these literature gaps, we aim to contribute to a more comprehensive understanding of the neurobiological basis of memory functions in ASD across the lifespan.

## 2 Methods

### 2.1 Participants

All the participants were selected from the open-access dataset, The Autism Brain Imaging Data Exchange (ABIDE I) (Di Martino et al., 2014) from multiple sites (USM, NYU, CALTECH, CMU, YALE, UM_1, UM_2, and SDSU) since these sites used the same parameters for repetition time (TR = 2s) and scanning sequence (interleaved). The initial sample was a total of 587 individuals with 280 in the ASD group and 307 in the Non-ASD group. To ensure the quality of the fMRI data, we only included participants: (a) with an age > 10 years; (b) with a full-scale IQ (FIQ) score above 70, and (c) with the framewise displacement of scans below 0.5 mm. After screening, there were 493 participants with 223 in the ASD and 270 in the Non-ASD groups. Using an in-house matching script, we then selected 75 participants with an ASD diagnosis and 75 Non-ASD individuals. We matched the gender, age, full-scale IQ, distribution across multiple sites, and their movement parameters (i.e., framewise displacement; FD) in the resting-state fMRI scan between the diagnosis groups (ASD vs. Non-ASD) and also across different age ranges (participants in the 10∼19, 20∼30, and 30+ age groups). The individuals in the Non-ASD group were not reported with other neurological or psychological disorders, whereas two autistic individuals were reported with ADHD, two with dysthymia, and five with anxiety, mood disorders, or phobia. After visual inspection of the imaging data, 2 participants from the ASD and 1 participant from the Non-ASD groups were excluded due to the low quality of the fMRI data. The detailed demographic information and the movement parameters of the final sample are presented in Table 1.

**TABLE 1:** Demographic, cognitive profiles, and movement parameters of matched age groups for ASD and Non-ASD groups.

		ASD	Non-ASD	*t*-value	*p*-value
All participants	n	73	74		
	F:M	7:66	10:64	χ^2^ = 0.24	0.627
	Age	23.05 (9.94)	22.99 (9.47)	0.04	0.971
	FIQ	105.93 (15.36)	109.85 (11.42)	−1.78	0.078
	FD	0.09 (0.07)	0.07 (0.05)	1.67	0.097
Age group (10–19)	n	30	30		
	F:M	2:28	4:26	χ^2^ = 0.19	0.667
	Age	14.2 (2.64)	14.3 (2.53)	−0.16	0.874
	FIQ	103.85 (17.38)	106.57 (13.26)	−0.68	0.499
	FD	0.09 (0.08)	0.08 (0.07)	0.74	0.463
Age group (20–29)	n	28	29		
	F:M	3:25	6:23	χ^2^ = 0.45	0.503
	Age	23.96 (3.17)	24.45 (2.98)	−0.62	0.537
	FIQ	107.07 (12.88)	111.83 (9.51)	−1.63	0.108
	FD	0.09 (0.06)	0.07 (0.05)	1.19	0.239
Age group (30 +: 30–56)	n	15	15		
	F:M	2:13	0:15	χ^2^ = 0.54	0.464
	Age	38.94 (6.79)	37.45 (7.2)	0.58	0.564
	FIQ	107.8 (16.2)	112.47 (9.97)	−0.95	0.35
	FD	0.09 (0.05)	0.07 (0.03)	1.43	0.164
Age group differences	Gender	χ^2^ = 0.58	χ^2^ = 3.62		
	Age	*F*(2, 70) = 194.76***	*F* (2,71) = 168.24***		
	FIQ	*F*(2, 70) = 0.46	*F*(2,71) = 2.15		
	FD	*F*(2, 70) = 0.095	*F*(2, 71) = 0.29		

F:M, female:male ratio; FSIQ, Full-scale IQ; FD, framewise displacement.

***p < 0.001.

### 2.2 fMRI processing and analysis

#### 2.2.1 Preprocessing

The resting-state fMRI data from ABIDE I were downloaded and analyzed using SPM12 (Ashburner et al., 2014). The first 8 volumes were not analyzed to allow for T1 equilibration. The data then were preprocessed with the standard pipeline, including correction for slice timing, motion realignment, and band filtering (0.008∼0.1 Hz). The individual data were then normalized into MNI152 space and then smoothed with a Gaussian kernel with a full width at half maximum (FWHM) of 6 mm.

#### 2.2.2 ROI Selection

We chose bilateral hippocampus (−/+24, −14, −20) for the hippocampal network of episodic memory (Kahn et al., 2008; Liu et al., 2023), and bilateral vTL (L: −45, −57, −15; R: 45, −36, −24) for the semantic memory based on previous literature (Visser and Lambon Ralph, 2011). In addition, we chose the following coordinates for the triple network nodes based on the literature (Uddin et al., 2011): for DMN, ventromedial prefrontal cortex (vmPFC), −2, 38, −12; posterior cingulate cortex (PCC), −6, −44, 34; for CEN, bilateral dorsolateral prefrontal cortex (dlPFC) −/+46, 20, 44 and bilateral posterior parietal cortex (PPC), −40, −56, 44, and 52, −52, 50; for SN, bilateral fronto-insular cortex (FIC) −34, 20, −8, and 39, 23, −4, and anterior cingulate cortex (ACC) 6, 24, 32. Note that these specific regions were chosen because previous literature suggested that they are critical to the dysfunctions in ASD (Nomi and Uddin, 2015), but these networks could encompass other brain regions that were not selected in this study, e.g., ventral parietal regions of DMN.

#### 2.2.3 ROI-to-ROI approach

The ROI-to-ROI network analysis was theory-driven to examine the relationship between memory networks and triple networks. At the individual level, the mean time series data of all ROI seeds (i.e., hippocampus, vTL, DMN, CEN, and SN seeds) were extracted, and Pearson’s correlation coefficients were calculated between all pairs of ROIs for each individual. We then examined the main effect of Group, Age, and the interaction between Age and Group on these coefficients across individuals with gender, FIQ, and FD entered as covariates. This analysis allowed us to reveal atypical functional connectivity between memory regions and brain regions that are important for ASD compared to Non-ASD based on previous literature (Uddin, 2015; Padmanabhan et al., 2017). Furthermore, we could also distinguish the different age effects between the groups.

#### 2.2.4 Seed to the whole brain approach

In addition to the theory-driven approach to examine the connectivity between memory seeds and regions in the triple networks, we also explored age-related effects beyond the triple networks. To be more conservative, we used a constrained approach to examine the age effects on functional connectivity in ASD. We examined the functional connectivity of hippocampal and vTL seeds that differed between the ASD and non-ASD groups first and then tested whether these patterns of connectivity showed age-related differences. At the individual level, the mean time series data of each hippocampal and vTL seed were de-meaned and then regressed on all other voxels in the brain for each participant while the effects of movement parameters on 6 directions (x, y, z, pitch, yaw, and roll) were controlled for. The beta coefficients were then converted to *t* maps for each ROI. At the group level, we compared the *t* -maps of autistic individuals to those of Non-ASD, to reveal the atypical functional connectivity in ASD. Following the recent literature (Abrams et al., 2019; Chen et al., 2022; Hoeppli et al., 2022; Tarchi et al., 2022), we first used a more relaxed threshold to examine the effects but also reported results at a more stringent threshold. At the relaxed level, the significant clusters were identified at a height threshold of *p* < 0.01 with family wise error (FWE) corrections for multiple comparisons at the cluster level of *p* < 0.01 (128 voxels based on Monte Carlo simulations). A more stringent height thresholding was carried out with *p* < 0.001 with a corrected cluster level of *p* < 0.01 (41 voxels). We then examined the main effect of Age and Age*Group interaction on all the revealed connectivity while controlling for the effects of gender, FIQ, and FD.

## 3 Results

### 3.1 ROI-ROI analysis

**FIGURE 1:**
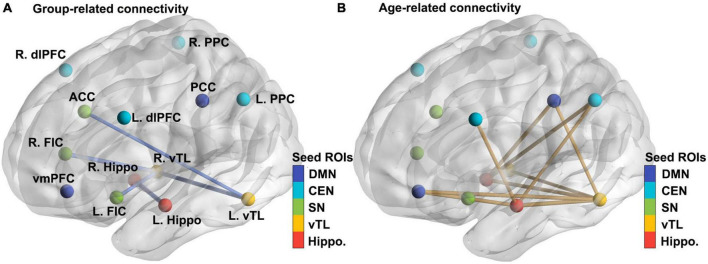
Significant effects of Group and Age on the functional connectivity between theory-driven ROIs chosen based on the literature. **(A)** Seed ROIs of five networks (hippocampal memory, ventral temporal memory, salience, central executive, and default-mode networks) and the group-related connectivity between them (all ASD < Non-ASD). The thickness of the lines represented the *t* values in the regression model. For more details, see Methods and Table 2. **(B)** The age-related connectivity between the same sets of theory-driven ROIs (reduced across ages). L/R dlPFC, left and right dorsolateral prefrontal cortex; PPC, posterior parietal cortex; ACC, anterior cingulate cortex; FIC, fronto-insular cortex; vmPFC, ventromedial prefrontal cortex; PCC, posterior cingulate cortex; hippo, hippocampus; vTL, ventral temporal lobe.

For hippocampal and vTL seeds, ASD overall showed reduced functional connectivity within the memory networks, between bilateral vTL and bilateral hippocampus seeds, as well as between left vTL and ACC, and right vTL and bilateral FIC (all *p*s < .05; see Table 2; Figure 1A) when the effects of gender, FIQ, and FD were controlled for. For age-related differences, we observed a general pattern of reduced connectivity over age in both ASD and Non-ASD groups (see Table 2; Figure 1B), and these connectivity patterns were found between hippocampal and vTL seeds, as well as the hippocampal and vTL seeds with DMN, CEN, and SN nodes. Interestingly, the functional connectivity showing the age-related effect and those showing the group-related effect had little overlap. However, we did not observe a reliable Age-by-Group interaction. At the uncorrected level, only the connectivity between the right vTL and the right hippocampus showed a significant interaction between Age and Group, β = −0.336, *t* = 2.235*, *p* = 0.027, but it did not survive the FDR correction, adjusted *p* = 0.108. In addition, when the two oldest participants were excluded, there was a significant age effect, β = −0.248, *t* = −2.373, *p* = 0.019, but with an attenuated Age-by-Group interaction, β = −0.230, *t* = −1.518, *p* = 0.1312.

**TABLE 2:** Significant results of main effects for Age and Group as well as the interaction between Age and Group in the ROI-ROI analysis.

Connectivity	Age	Group	Age*Group
L. Hippo—R. Hippo	β = −0.193, *t* = −1.744^#^	β = 0.424, *t* = 2.642**	β = 0.007, *t* = 0.042
L. Hippo—L. vTL	β = −0.270, *t* = −2.513*	β = 0.255, *t* = 1.638	β = −0.176, *t* = −1.139
L. Hippo—L. dlPFC	β = −0.235, *t* = −2.138*	β = 0.111, *t* = 0.697	β = −0.009, *t* = −0.578
L. Hippo—L. PPC	β = −0.218, *t* = −1.988*	β = 0.175, *t* = 1.100	β = −0.157, *t* = −0.996
L. Hippo—vmPFC	β = −0.245, *t* = −2.241*	β = 0.215, *t* = 1.353	β = −0.081, *t* = −0.516
R. Hippo—L. vTL	β = −0.241, *t* = −2.249*	β = 0.185, *t* = 1.186	β = −0.221, *t* = −1.432
R. Hippo—L. PPC	β = −0.247, *t* = −2.272*	β = 0.104, *t* = 0.658	β = −0.167, *t* = −1.072
R. Hippo—PCC	β = −0.249, *t* = −2.301*	β = 0.242, *t* = 1.539	β = −0.010, *t* = −0.629
L. vTL—PCC	β = −0.222, *t* = −2.017*	β = 0.237, *t* = 1.487	β = −0.104, *t* = −0.658
L. vTL—vmPFC	β = −0.251, *t* = −2.282*	β = 0.153, *t* = 0.960	β = −0.108, *t* = −0.685
L. vTL—R. vTL	β = −0.223, *t* = −2.025*	β = 0.397, *t* = 2.483*	β = −0.034, *t* = −0.220
L. vTL—ACC	β = −0.083, *t* = −0.744	β = 0.323, *t* = 1.994*	β = −0.203, *t* = −1.265
R. vTL—L. FIC	β = −0.065, *t* = −0.600	β = 0.334, *t* = 2.112*	β = −0.288, *t* = −1.841^#^
R. vTL—R. FIC	β = −0.032, *t* = −0.296	β = 0.408, *t* = 2.569*	β = −0.284, *t* = −1.808^#^
R. vTL—R. Hippo	β = −0.194, *t* = −1.853^#^	β = 0.153, *t* = 1.008	β = −0.336, *t* = 2.235*

Hippo, hippocampus; vTL, ventral temporal lobe; vmPFC, ventromedial prefrontal cortex; dlPFC, dorsolateral prefrontal cortex; FIC, fronto-insular cortex; PPC, posterior parietal cortex; PCC, posterior cingulate cortex.

^#^*p* < 0.10;

*p < 0.05;

**p < 0.01.

### 3.2 Seed-based analysis

**FIGURE 2:**
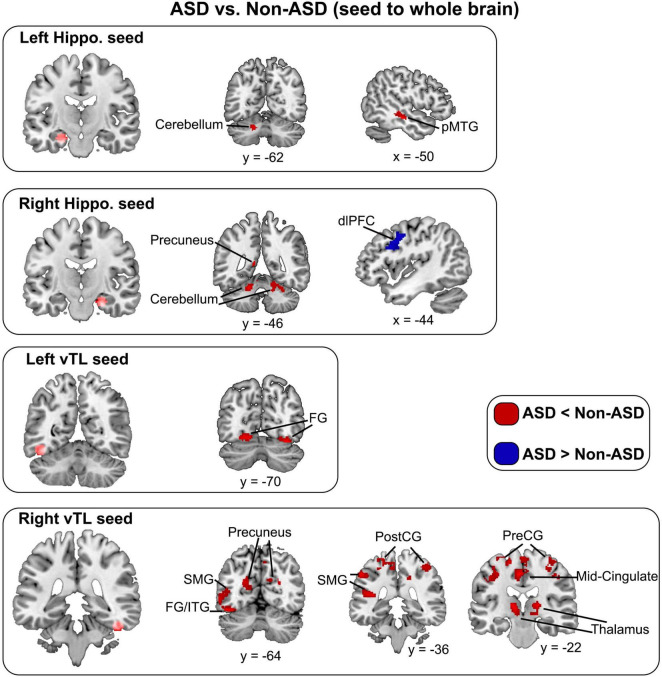
Seed to the whole brain analysis showed the atypical connectivity (ASD < Non-ASD in Red and ASD > Non-ASD in Blue) of bilateral hippocampal and vTL seeds. Hippo, hippocampus; pMTG, posterior middle temporal gyrus; vITG, ventral inferior temporal gyrus; FG, fusiform gyrus; dlPFC, dorsolateral prefrontal cortex; SMG, supramarginal gyrus; PostCG, postcentral gyrus; PreCG, precentral gyrus.

We further explored the age-related effects in functional connectivity of hippocampal and vTL memory seeds beyond the triple networks. We observed that overall, the ASD group showed reduced functional connectivity of both hippocampus and vTL, but a greater connectivity of the right hippocampus with the left dorsolateral prefrontal cortex compared to Non-ASD (see Table 3; Figure 2). We did not find any significant relationship between the Age and the functional connectivity of hippocampal or vTL seeds or a significant interaction between Age and Group on atypical connectivity of ASD compared to Non-ASD.

**TABLE 3:** Brain regions showing significantly different functional connectivity of hippocampal and vTL seeds in ASD compared to Non-ASD.

Cluster	Hemisphere	Region	Peak center in MNI space (x, y, z)	Cluster size (mm^3^)	*t*-value
**Left hippocampus (ASD < Non-ASD)**					
1	Left	posterior MTG	−50, −22, −12	177	3.55
2	Left	Cerebellum	−18, −62, −30	138	3.2
**Right hippocampus (ASD < Non-ASD)**					
1	Left	Precuneus	−12, −46, 10	261	2.91
2	Right	Cerebellum	8, −72, −34	1,387	3.41
**Right hippocampus (ASD > Non-ASD)**					
1	Left	dlPFC	−44, 2, 44	380	3.16
**Left vTL (ASD < Non-ASD)**					
1	Left	Fusiform gyrus	−16, −72, −12	345	3.35
2	Right	Fusiform gyrus	32, −70, −18	275	3.62
**Right vTL (ASD < Non-ASD)**					
**1**	**Left**	**FG/ITG**	−**46,** −**54,** −**22**	**1,156**	**4.33**
**2**	**Left**	**Postcentral gyrus**	−**36,** −**18, 38**	**1,930**	**4.21**
3	Left	Precentral gyrus	0, −14, 68	550	3.93
**4**	**Left**	**Thalamus**	−**14,** −**26, 6**	**226**	**3.92**
5	Left	Middle cingulate cortex	−4, −22, 48	186	3.84
6	Left	Supramarginal gyrus	−54, −36, 46	424	3.73
7	Left	Precuneus	−18, −68, 22	338	3.71
8	Right	Postcentral gyrus	12, −44, 56	261	3.6
9	Right	Thalamus	18, −22, 8	139	3.54
10	Right	Precentral gyrus	52, −4, 30	190	3.36
11	Right	Postcentral gyrus	34, −26, 56	314	3.33
12	Right	Precuneus	26, −64, 22	183	3.28

MTG, middle temporal gyrus; dlPFC, dorsolateral prefrontal cortex; FG/ITG, fusiform gyrus/inferior temporal gyrus. The bolded regions also survived a more stringent threshold with *p* < 0.001 with a cluster size of 41.

## 4 Discussion

Our study used a well-matched sample of individuals with ASD and Non-ASD across a broad age spectrum and unveiled significant associations between resting-state functional connectivity within the hippocampal and ventral temporal lobe (vTL) memory networks. Specifically, both memory networks showed a notable reduction in connectivity with nodes in the triple networks over aging. Surprisingly, no reliable difference in age-related effects was observed in ASD compared to Non-ASD individuals. These observations urged further research to focus on the neurobiological basis of the memory and cognitive challenges experienced by autistic individuals.

Overall, both our ROI-to-ROI and seed-based analyses demonstrated reduced resting-state functional connectivity of the hippocampal and vTL networks in ASD compared to Non-ASD individuals. The ROI-to-ROI analysis mostly revealed the reduced connectivity within the hippocampal and vTL memory networks as well as the bilateral vTL with the SN nodes, namely ACC and bilateral FIC. Surprisingly, the seed-based analysis revealed little overlaps but identified a few more regions beyond the triple networks. These results aligned with findings from some previous studies (Lynch et al., 2013; von dem Hagen et al., 2013; Verly et al., 2014; Eilam-Stock et al., 2016; Arnold Anteraper et al., 2019), but not others (Mennes et al., 2011; Liu et al., 2023). Hence, our results further underscored that dysfunctions in ASD relate to a complex pattern of both greater and reduced intrinsic signals between different brain regions (Yizhar et al., 2011; Uddin et al., 2013; Kleinhans et al., 2016; Foss-Feig et al., 2017; Hull et al., 2017). Several prior investigations have specifically explored the resting-state functional connectivity of the hippocampus (Nomi and Uddin, 2015; Traynor et al., 2018; Liu et al., 2023) and found greater hippocampal connectivity in ASD compared to their control groups. One plausible explanation could be that the autistic participants in these studies were predominantly in the younger age range (<20 years old), consistent with our findings indicating a reduction in the functional connectivity of the hippocampal memory network with age in autistic individuals. Given the limited understanding of vTL-based functional connectivity in ASD, our study suggests that challenges in semantic processing (Ehlen et al., 2020; Foldager et al., 2023) may be associated with reduced connections of the vTL with other brain regions such as the dorsolateral prefrontal cortex, fronto-insular cortex, and hippocampus, which play crucial roles in information encoding and retention (Rosenbaum et al., 2001; O’Reilly and Norman, 2002; Postle, 2009; Dickerson and Eichenbaum, 2010), as well as with motor-sensory regions such as the fusiform gyrus and pre/postcentral gyri, involved in processing and representing multi-modal information (Rogers et al., 2005; Pulvermuller et al., 2009; Binkofski and Buxbaum, 2013; Chen and Rogers, 2015; Lambon Ralph et al., 2016; Bi, 2021).

Our study also revealed reduced functional connectivity of bilateral hippocampal and left vTL seeds over aging in ASD, compared to Non-ASD individuals. This finding may help reconcile some inconsistencies in the literature regarding both greater and reduced functional connectivity in ASD (Abrams et al., 2013; Verly et al., 2014; Hull et al., 2017; Lynn et al., 2018; Traynor et al., 2018; Liu et al., 2023), suggesting that participants’ age may be a contributing factor. Additionally, our findings may imply a nonlinear relationship between age and functional connectivity of memory networks in ASD, as indicated by structural and functional differences reported in previous research (Reinhardt et al., 2020; Pagni et al., 2022). However, one limitation is that the sample size of older adult participants was relatively small (15 ASD and 15 non-ASD), possibly affecting the accuracy of our estimations regarding age-related effects. Therefore, future studies should aim to recruit larger cohorts of elderly adults to further explore potential differences between ASD and Non-ASD groups.

Notably, the age-related effects were observed in the ROI-to-ROI approach, but not in the seed-based whole-brain analysis. One possible explanation could be that the age-related differences in functional connectivity are more likely to be observed between the memory networks, especially the hippocampal regions, and brain regions in the triple networks. Another reason is pertinent to the method we used in the seed-based whole-brain analysis. To be more conservative with our approach, we only examined the age-related effects in the brain regions that showed significant differences between the ASD and Non-ASD groups. However, from the ROI-to-ROI results, we observed that the age-related and group-related effects hardly overlapped. If the age-related effects on functional connectivity are relatively independent of differences between ASD and Non-ASD groups in brain regions beyond the triple networks as well, our seed-based whole brain analysis was less likely to reveal significant age-related effects. Therefore, future studies should examine the age-related effects independently.

One of the most important goals of the current study is to examine the connectivity difference in age-related effects between ASD and Non-ASD groups. To our surprise, we did not reveal any reliable Age-by-Group interaction. We only observed a potential trend of less pronounced reduction in functional connectivity between the right hippocampus and vTL in ASD. However, this finding was not significant after multiple comparison corrections and the exclusion of possible outliers. The right hippocampus has been shown to exhibit atypical developmental trajectories in ASD (Schumann, 2004; Reinhardt et al., 2020), with its abnormalities linked to social and cognitive challenges in ASD (Hogeveen et al., 2020; Liu et al., 2023). Meanwhile, the right ventral temporal lobe has been associated with challenges in face recognition (Schultz et al., 2000; Scherf et al., 2015), symptom severities (Kim et al., 2021), and language processing (Graves et al., 2022) in ASD. Therefore, given the significance of these two regions in ASD, although our study did not reveal reliable effects for these two regions, it is then critical for the future study to examine the atypical connectivity between the hippocampal and vTL memory networks, and their roles in in memory, language, and social challenges in autistic individuals.

Our study has clear limitations. First, we did not examine the relationship between atypical functional connectivity and memory abilities in ASD. Thus, it remains unclear how reduced connectivity in ASD may be associated with their social and cognitive abilities over aging. Furthermore, our study was based on a publicly available dataset and we tried to carefully match the ASD and Non-ASD groups, and therefore, the number of participants we included was still small, and the individuals in the older age range were limited (only 15 in each group with an age larger than 30). Consequently, the effect of the Age-by-Group interaction was relatively weak, which was only observed in a theory-driven and planned ROI-to-ROI analysis. Also, it did not survive with a correction for multiple corrections, or when the older participants were excluded from the analysis. Future studies should utilize a much larger sample with more participants in the older age range. This also highlighted the inadequacy of the current research to examine aging in ASD.

In conclusion, our study provides evidence of reduced functional connectivity in the hippocampal and vTL memory networks in ASD across the lifespan, emphasizing the importance of investigating memory network dysfunctions in ASD to better understand cognitive challenges in memory, language, and social skills. Furthermore, our results offer insights for educational and clinical practices to address both episodic and semantic memories when supporting autistic individuals in achieving independence in various aspects of life. Future research should delve deeper into how the atypical connectivity of memory networks may be linked to various types of memory challenges across different age groups in ASD.

## Data availability statement

Publicly available datasets were analyzed in this study. This data can be found at: ABIDE I (https://fcon_1000.projects.nitrc.org/indi/abide/).

## Ethics statement

Ethical approval was not required for the study involving humans in accordance with the local legislation and institutional requirements. Written informed consent to participate in this study was not required from the participants or the participants’ legal guardians/next of kin in accordance with the national legislation and the institutional requirements.

## Author contributions

LC: Conceptualization, Formal analysis, Investigation, Methodology, Project administration, Supervision, Visualization, Writing – original draft, Writing – review & editing. MA: Visualization, Writing – original draft, Writing – review & editing. MF: Visualization, Writing – original draft, Writing – review & editing. YG: Visualization, Writing – original draft, Writing – review & editing. ZT: Visualization, Writing – original draft, Writing – review & editing. XZ: Writing – original draft, Writing – review & editing.
